# Knowledge, attitudes and practices of contraceptive methods among women seeking voluntary termination of pregnancy at Jubilee Hospital, Pretoria, South Africa

**DOI:** 10.4102/phcfm.v11i1.1919

**Published:** 2019-08-15

**Authors:** Tombo Bongongo, Indiran Govender

**Affiliations:** 1 Department of Family Medicine, Sefako Makgatho Health Sciences University, Pretoria, South Africa

**Keywords:** knowledge, attitudes, practices, contraceptive methods, voluntary termination of pregnancy

## Abstract

**Background:**

There is a high rate at which women in South Africa who are of childbearing age and still opt for abortions or voluntary termination of pregnancy (VTOP). Despite the availability of free contraceptive methods and health education in all health facilities across the country, to reduce and prevent unwanted pregnancies and VTOP, there is still an alarming increase in the rate of VTOP.

**Aim:**

This study sought to determine the knowledge, attitudes and practices of contraceptive methods among women seeking VTOP.

**Setting:**

The study was conducted at Jubilee Hospital, Pretoria, South Africa.

**Methods:**

A cross-sectional survey was taken using a piloted, structured and self-administered questionnaire. Convenience sampling was applied and the sample size was 126.

**Results:**

The mean age of the 126 participants was 26.1 years. Findings obtained after analysis of participants’ data were grouped following the university categorisation. A score below 50% was referred to as a ‘poor’ outcome that from 50% to 74% was referred to as a ‘satisfactory’ outcome and that beyond 74% was considered as an ‘excellent’ outcome. Knowledge was poor for 28 (22.2%) women. It was satisfactory for 91 (72.2%) women and excellent for 7 (5.5%) women. Looking at the attitude: 124 (98.4%) approved the use of contraception, 1 (0.79%) disapproved and 1 (0.79%) abstained because of religious beliefs. Regarding the practice of contraception: 92 (73.0%) have already used contraceptive methods, while 34 (27.0%) have not.

**Conclusion:**

In summary, the study showed a satisfactory knowledge of contraceptive methods, a positive attitude towards contraception and a huge number of participants who had already used contraceptive methods, among women seeking VTOP at Jubilee Hospital, Pretoria, South Africa.

## Introduction

In South Africa, the *Choice on Termination of Pregnancy Act of 1996*, passed in 2008, allows pregnant women to terminate their pregnancies. There are 260 000 termination of pregnancies (TOPs) performed every year in South Africa; almost half of them are illegal. This alarmingly high rate of voluntary termination of pregnancy (VTOP) is noted despite the government’s efforts to not only educate people but also provide the necessary contraceptive measures in order to prevent unwanted pregnancies.^1^ The South African VTOP rates have also increased between 2010 and 2012.^1^ At Temba Community Health Centre (CHC), there is an increasing trend of women requesting termination of their pregnancies. Many of the women are referred to the nearby Jubilee District Hospital where approximately 32 VTOPs are performed monthly.^2^

Unplanned pregnancies constitute a public health issue worldwide. Existing reliable and accessible methods of contraception could have resolved the issue of unplanned pregnancies and the increase in unplanned pregnancies can be attributed to the lack of literature addressing the knowledge of women regarding contraceptive measures. This probable lack of knowledge could explain the high rates of abortion that have been reported in numerous countries in the world. While assessing the knowledge of women on contraceptive measures, through a survey that was conducted in the United States (US), a wrong belief about the effectiveness of condoms was captured. This observation of a wrong belief implied that much has to be done about educational opportunities and research on contraceptive measures because these strategies will empower women regarding contraceptive methods.^3^

To reach low rates of abortion and give space to contraceptive measures, a national US survey, considering data from 1982, 1988 and 1995, showed that the target can be achieved while addressing the risky behaviour, encouraging the use of contraceptive measures and ensuring the efficacy of the chosen contraceptive methods.^4^ Abortion is performed in the USA as a result of multiple, diverse and interrelated reasons such as the child interfering with a woman’s life, financial constraints, a woman in a difficult relationship, a lack of support and other reasons.^4^

Contrary to the US, assessing knowledge, attitudes and practices on contraceptive measures raise legal equivoques in some countries, such as Botswana. Women abort clandestinely only because the law does not allow abortion and many of the women then experience complications that result in a hysterectomy and even death.^5^ In other African countries, abortion is legally practised but the level of knowledge regarding contraceptive measures remains questionable. Women have little and incorrect information on reproductive biology, as they rely on information provided to them by their schoolmates and the media. This lack of knowledge has been verified by the fact that young urban Nigerian women are not aware that the first intercourse can lead to pregnancy and even fewer know that the pregnancy risk varies during the menstrual cycle.^6^

Little knowledge about contraceptive measures may also be occasioned by certain religious and cultural beliefs, as noted in the East of the Democratic Republic of Congo (DRC). With an increasing rate of HIV and AIDS, students in this part of the DRC are aware of condoms but because of their beliefs, they seldom used the condoms.^7^

The awareness of contraceptive measures was much higher in educative environments such as schools and universities where contraception was part of the curriculum. In Lesotho, where a high level of sexual experience was recorded among students, the awareness and utilisation of contraceptive measures were high and assessed to be 97.5%; this was the result of the educational programme applied among students.^8^ This strategy of incorporating contraceptive information could have also assisted in rural KwaZulu-Natal where adolescents had a high level of sexual activity but low contraceptive use^9^ as well as in the Eastern Cape where adolescents were sexually active but had insufficient information and harboured misunderstandings on contraception.^10^ A good awareness versus low use of contraceptives led to a high rate of unwanted pregnancies in the Lower Umflozi District in South Africa.^11^ In 2015, abortion was studied in the same Jubilee Hospital but focused on the characteristics of women seeking abortion. Most of the users of the TOP clinic who had a low level of education were not working, were under parental care or were widows or single. They experienced economic-financial challenges and others were in difficult relationships.^2^

Looking at the problem of the high incidence of request for TOP, as presented in the literature, globally and nationally, much has to be done in order to reduce the rate of VTOP. In support of that, this study sought to determine the knowledge, attitudes and practices of contraceptive methods, among women seeking VTOP in Jubilee Hospital, Pretoria.

## Method

### Study design

This was a descriptive cross-sectional survey using a piloted, structured and self-administered questionnaire.

### Setting

The study was conducted at Jubilee Hospital. It is one of the district hospitals located in the North of the Gauteng province. The hospital has 551 beds and the catchment area constitutes roughly 7.5 million in population. It is surrounded by 11 clinics, none of which renders the VTOP services. They refer their clients to the Jubilee District Hospital.

### Study population and sampling

All clients who attended the TOP clinic at the Jubilee District Hospital from the 01 August 2014 up to 28 February 2015 were considered for this study. Clients younger than 18 years of age were excluded from the study. The levels of maturity and confidentiality of the participants, as it was a self-administered questionnaire, were considered. Considering the age, the total number of clients who presented themselves at the clinic for VTOP during the data collection period was 129. After explanation of the study and its purposes, three participants did not consent to the study. No sample size calculation was applied because all of the 126 clients were considered as the sample.

### Data collection

A trained research assistant provided an explanation about the study to the clients seeking VTOP. The 126 clients who consented to participate in the study were given the questionnaire by the research assistant. The questionnaire was developed by the researcher and then tested through a pilot study conducted at Odi District Hospital in Soshanguve. Odi is the only district hospital in sub-district 1, while Jubilee is the only district hospital in sub-district 2. The sub-districts are neighbours and have people with many similarities such as language, eating habits, religious and cultural beliefs. The pilot took place at Odi Hospital in order to avoid contaminating data. The questionnaire was reviewed by the second author who is an expert. From the outcome of the pilot study and the review done by the expert, the questionnaire was reformulated, based on the objectives of the study, before it was used at the Jubilee Hospital.

### Data analysis

Data were captured and stored on a Microsoft Excel spreadsheet. It was then analysed using Statistical Package for Social Sciences (SSPS) software version 9.2. Descriptive statistics was used to analyse the data. A *p* value of 0.05 was considered significant. The findings obtained after analysis of the clients’ knowledge of the different types of contraception were classified as follows: a score lower than 50% was regarded as a ‘poor’ outcome, a score from 50% to 74% was referred to as ‘satisfactory’ and a score above 74% was considered as ‘excellent’. This is a copy of how students have been classified at the university by the exam committee. In analysing the attitude, satisfaction and readiness to use contraception was considered as a ‘positive attitude’, while a refusal to consider and use contraception was considered as a ‘negative attitude’ towards contraception. For contraceptive practice, the study differentiated clients who had used contraceptive methods and those, still using contraception against those who had never used it.

### Ethical considerations

Ethical approval was obtained from the Sefako Makgatho Research Ethic Committee (SMUREC) (Clearance number: MREC/M/280/2014: IR) and the CEO of the Jubilee Hospital. Throughout the research process, the ethical principles of confidentiality, anonymity, beneficence and non-maleficence were maintained. The care of the client at the hospital was not compromised whether she agreed to participate or not in the study.

## Results

### Demographic characteristics of the clients

The overall mean age of the 126 participants was 26.1 years, with a standard deviation of 6.55 years. Most of the women (45 or 35.7%) were in the age group of 20–24 years (see Table 1). The majority of the women (93.7%, 118) were single. The majority of the women (103, 81.75%) had reached the secondary level of education. Most of the women (101, 80.16%) were unemployed.

**TABLE 1:** Demographic characteristics of the women.

Demographic characteristic	Number of women	Percentage
**Age groups**		
< 20 years	18	14.29
20–24 years	45	35.71
25–29 years	26	20.63
30–34 years	20	15.87
35–39 years	12	9.52
40–44 years	05	03.97
**Marital status**		
Divorced	1	0.1
Married	7	5.56
Single	118	93.65
**Level of education**		
None	1	0.79
Primary	5	3.97
Secondary	103	81.75
Tertiary	17	13.49
**Employment**		
Unemployed	101	80.16
Employed	25	19.84
**Total**	**126**	**100**

### Knowledge of different contraceptive methods

To assess the knowledge, participants answered ‘yes’ or ‘no’ about whether they are aware of the different contraceptive methods, as presented in Table 2. The most known contraceptive method was the male condom (98.4%) followed by the female condom (96.8%) and then pill (96%) and implanon (82.9%).

**TABLE 2:** Awareness of the different contraceptive methods.

Question: Are you aware of these contraceptive methods?	Yes		No	
	Frequency	Percentage	Frequency	Percentage
Pill	121	96.00	5	3.97
Sterilisation	88	69.80	38	30.20
Male condom	124	98.40	2	1.60
Female condom	122	96.80	4	3.20
Loop	92	73.00	34	26.90
Vasectomy	26	20.60	100	79.40
Withdrawal	100	79.37	26	20.60
Vaginal ring	8	6.40	118	93.70
Patch	14	11.10	112	88.90
Implanon	107	82.90	19	15.10
Calendar	26	20.63	100	79.40

Twenty-eight participants (22.2%) scored less than 50% on the knowledge questions and were categorised as having poor knowledge (see Table 3).

**TABLE 3:** Knowledge of contraceptive methods.

Knowledge of types of contraceptive methods	Number of women	Percentage
Poor (< 50%)	28	22.2
Satisfactory (50%–74%)	91	72.2
Excellent (> or = 75%)	7	5.5
**Total**	**126**	**100.0**

#### Sources of contraceptive knowledge

Most clients 77 (61.1%) obtained their contraceptive information from health care professionals, while 11 (8.7%) got their knowledge through the media (such as radios, newspapers and television) and 38 (30%) obtained this information from their social contacts (family and friends).

### Attitudes about contraceptive methods

To assess attitude, participants answered one question by indicating whether they approved, disapproved and any other attitude which they were encouraged to label.

There were 124 (98.4%) clients who approved to use contraceptive methods, one disapproved and one for religious concerns did not want to comment (see Table 4).

**TABLE 4:** Attitude towards contraceptive measures.

Attitude towards contraceptive methods	Number of women	Percentage
Approved	124	98.4
Disapproved	1	0.79
Religious beliefs	1	0.79
**Total**	**126**	**100.00**

**TABLE 5:** Reasons for using and stopping contraceptive methods.

Variable	Frequency	Percentage
**Already used contraceptive methods**		
Yes	92	73.0
No	34	26.9
**Reasons for using contraceptive methods**		
Economical	18	19.6
Family pressure	24	26.1
Improvement of health	8	8.7
Spacing births	9	9.8
Other reasons	33	35.9
**Stopped using contraceptive methods**		
Bleeding	38	45.2
Did not like the method	15	17.9
Wanted to get a child	19	22..6
Other reasons	9	10.7
No reasons	3	3.6

### Practices of contraceptive methods

Seventy-three per cent of women or participants had already used contraceptive methods, while 27 declined.

Except for other reasons (35.9%) that were not revealed by the participants, family pressure (24%) followed by economic reasons (19.6%), then spacing births and improvement of health (8.7%) were the reasons that led to the practice of contraception in Hammanskraal.

Some of the women stopped contraception because of the adverse effects such as bleeding (45.2%), desire for a child (22.6%) or did not like the contraceptive method any longer (17.9%).

## Discussion

Despite the availability of free contraceptives and health education in all the health facilities across South Africa, to reduce and prevent unwanted pregnancies, and to reduce the rate of VTOP in the country, the necessity of educating women seems to be one of the ways that will empower women. According to more than one researcher, educating women on contraception could reduce the practice of VTOPs in the US^3^ as well as in Hammanskraal, Pretoria. The bar has to be raised from a satisfactory knowledge, 72.2% as demonstrated in this current study, to a hundred percent knowledge of contraception.

Women can be better educated on contraceptive measures and acquire enough knowledge and a positive attitude towards contraception. The applicability of women’s knowledge and attitudes towards TOP are sometimes restricted because of some ethical and legal issues, such as is the case in Botswana. This explained why women opted for a clandestine TOP even with possible negative consequences.^5^

The importance of education on contraceptive measures still has its place in many countries of the world. Through education, misconceptions will be corrected and new knowledge will be acquired; this can positively influence the attitudes and the practices of contraception, as in the case of young urban Nigerians.^6^

Education can be used as a weapon to spread knowledge through communities and can face difficulties, when opposed to cultural and religious beliefs. In the East DRC, there was little knowledge about the use of condoms because of certain beliefs and this reduced the use of condoms in the region.^7^

The high awareness and prevalence of contraceptive methods used among students at the University of Lesotho, related to the high level of sexual experience, showed that teaching family planning in their schools contributed to a positive impact while targeting the reduction of VTOPs. In Hammanskraal, the level of knowledge regarding contraceptive methods was 72.2%, while in Lesotho it was 97.5%. This highlighted the fact that an educational environment that includes information on contraception improves the knowledge, attitudes and even the practices. Knowledge can play a great role while targeting to reduce the rate of VTOPs in the country. This can be recommended for rural KwaZulu-Natal^9^ as well as for the Eastern Cape, where a high level of sexual activity but low contraceptive use has been reported.^10^ To reduce the number of VTOPs, Hammanskraal should also embark on educating its people, especially in schools and colleges.

In the Lower Umflozi District of South Africa, an outcome similar to that of Hammanskraal was found regarding the level of knowledge about contraceptive methods. Both studies concluded that a good knowledge of contraceptive methods existed among women. Looking at the statistics of abortion in the country, women need continuous education on contraceptive methods because this may reduce the rate of VTOP.^11^

The study conducted by Ndwambi and Govender^2^ at the Jubilee Hospital on abortion and the current study had some similarities that need to be noted. Both studies have shown that young, single and unemployed women were the most vulnerable, who required abortion in Hammanskraal. As a remedy, this category of women should be the target when organising education on contraception to empower them in terms of knowledge, attitudes and practices and ultimately reduce the rate of the VTOP in the area.^2^

## Limitations

Besides the duration of the data collection period, and the fact that the study did not consider all age groups exposed to VTOP, considered only women 18 years and above, and also consented to participate in the study, the results may not be representative of the entire Hammanskraal community, but this study presents one of the health issues of this community.

## Conclusion and recommendations

The women in this study, seeking TOP, demonstrated a satisfactory knowledge of and a positive attitude towards contraceptive methods. Most of the women had used contraception previously. However, knowledge, attitudes and practices of contraceptive methods among women in Hammanskraal have to be increased in order to reduce unplanned pregnancies and thus the rate of VTOP. Knowledge has to be passed to the community by health care professionals, as most women obtain information on family planning and contraceptive methods from these professionals and seem to trust the knowledge of health care professionals.

Besides medical practitioners, information can be spread using continuous education by health talks in the hospital, by professional nurses in the surrounding clinics, schools and colleges and by community health workers through the ward-based outreach team programme.
